# Propofol-induced Unresponsiveness Is Associated with a Brain Network Phase Transition

**DOI:** 10.1097/ALN.0000000000004095

**Published:** 2022-02-08

**Authors:** Rebecca M. Pullon, Catherine E. Warnaby, Jamie W. Sleigh

**Affiliations:** From the Department of Anesthesiology, Faculty of Medical and Health Sciences, University of Auckland, Auckland, New Zealand (R.M.P., J.W.S.); the Wellcome Center for Integrative Neuroimaging and Nuffield Division of Anaesthetics, Nuffield Department of Clinical Neurosciences, University of Oxford, Oxford, United Kingdom (C.E.W.).

## Abstract

**Background:**

The wakeful brain can easily access and coordinate a large repertoire of different states—dynamics suggestive of “criticality.” Anesthesia causes loss of criticality at the level of electroencephalogram waveforms, but the criticality of brain network connectivity is less well studied. The authors hypothesized that propofol anesthesia is associated with abrupt and divergent changes in brain network connectivity for different frequencies and time scales—characteristic of a phase transition, a signature of loss of criticality.

**Methods:**

As part of a previously reported study, 16 volunteers were given propofol in slowly increasing brain concentrations, and their behavioral responsiveness was assessed. The network dynamics from 31-channel electroencephalogram data were calculated from 1 to 20 Hz using four phase and envelope amplitude–based functional connectivity metrics that covered a wide range of time scales from milliseconds to minutes. The authors calculated network global efficiency, clustering coefficient, and statistical complexity (using the Jensen–Shannon divergence) for each functional connectivity metric and compared their findings with those from an *in silico* Kuramoto network model.

**Results:**

The transition to anesthesia was associated with critical slowing and then abrupt profound *decreases* in global network efficiency of 2 Hz power envelope metrics (from mean ± SD of 0.64 ± 0.15 to 0.29 ± 0.28 absolute value, *P* < 0.001, for medium; and from 0.47 ± 0.13 to 0.24 ± 0.21, *P* < 0.001, for long time scales) but with an *increase* in global network efficiency for 10 Hz weighted phase lag index (from 0.30 ± 0.20 to 0.72 ± 0.06, *P* < 0.001). Network complexity decreased for both the 10 Hz hypersynchronous (0.44 ± 0.13 to 0.23 ± 0.08, *P* < 0.001), and the 2 Hz asynchronous (0.73 ± 0.08 to 0.40 ± 0.13, *P* < 0.001) network states. These patterns of network coupling were consistent with those of the Kuramoto model of an order–disorder phase transition.

**Conclusions:**

Around loss of behavioral responsiveness, a small increase in propofol concentrations caused a collapse of long time scale power envelope connectivity and an increase in 10 Hz phase-based connectivity—suggestive of a brain network phase transition.

## Abstract 2

Temporospatial electroencephalographic analysis of brain network dynamics over a wide range of frequencies and time scales in 16 volunteers receiving slowly increasing concentrations of propofol revealed that transition to unresponsiveness was associated with a sudden rise in alpha frequency network phase synchrony anteriorly, but also a transient surge and then loss of network coupling over long (tens of seconds) time scales. Deep anesthesia was characterized by alpha waveform hypersynchrony and slow-wave power envelope dissynchrony across the whole cortex. These observations suggest that propofol anesthesia is associated with a constellation of changes in network connectivity across frequencies and time scales that are signatures of sharp and sudden transitions in the behavior of networks.

**Editor’s Perspective**

**What We Already Know about This Topic**

Changes in levels of consciousness are closely linked to anesthetics-induced dynamic alterations in functional connectivity of the central nervous system

Propofol induces consistent global and regional decreases in functional brain connectivity when measured over the time scale of seconds to tens of seconds

The question of how propofol affects neuronal network dynamics during both shorter (milliseconds) and longer (minutes) periods is incompletely explored

**What This Article Tells Us That Is New**

Temporospatial electroencephalographic analysis of brain network dynamics over a wide range of frequencies and time scales in 16 volunteers receiving slowly increasing concentrations of propofol revealed that transition to unresponsiveness was associated with a sudden rise in alpha frequency network phase synchrony anteriorly, but also a transient surge and then loss of network coupling over long (tens of seconds) time scales

Deep anesthesia was characterized by alpha waveform hypersynchrony and slow-wave power envelope dissynchrony across the whole cortex

These observations suggest that propofol anesthesia is associated with a constellation of changes in network connectivity across frequencies and time scales that are signatures of sharp and sudden transitions in the behavior of networks

Propofol causes alterations in large-scale regional brain functional connectivity/coupling, which seem to be closely linked to changes in level of consciousness, as reviewed by Mashour and Hudetz^1^ and Lee and Mashour.^2^ When functional magnetic resonance imaging is used to measure functional connectivity during longer time scales (seconds to tens of seconds), propofol induces consistent global and regional decreases in connectivity.^3,4^ However, when connectivity is measured over shorter time scales (milliseconds to seconds) using electroencephalogram (EEG), the situation is less clear cut, and a number of discordant observations have been reported. Some groups suggest that connectivity decreases are minimal, or regionally localized.^5^ Conversely, a profound *increase* in frontal alpha (8 to 12 Hz) frequency band coherence with anesthesia has been widely observed.^6,7^ Thus, the same anesthetic intervention is apparently simultaneously causing uncoupling, and also coupling, between brain regions—depending on the choice of frequencies, time scales, and metrics used. As a result, the underlying biologic explanations have tended to be somewhat *ad hoc* and focused on isolated EEG phenomena. Is there a single underlying process that could explain these diverse observations?

The concept of brain criticality could be one such unifying explanatory framework. Phase transitions correspond to sharp and sudden changes of the behavior of systems. For more than a decade, it has been suggested that the network dynamics of the wakeful brain self-organize to lie close to a network phase transition point—in a zone of so-called “extended dynamical criticality”—that enables the brain to easily access the large repertoire of states needed for conscious wakefulness.^8^ There are various ways to infer the existence of criticality. Commonly, investigators seek to demonstrate the existence of power-law exponents in the brain’s spatiotemporal dynamics.^9,10^ This has been criticized because it is possible to replicate these results without involving any criticality.^11,12^ Another approach is to quantify the spatiotemporal complexity of the brain by the eigenmodes of autocorrelation models fitted to multielectrode data.^13,14^ These authors showed that anesthesia is associated with stabilization of brain dynamics, indicating of loss of criticality. We pursued a third avenue of investigation based on the fact that if the awake brain lies in a critical dynamical region, we should be able to precipitate the associated nearby network order–disorder phase transition by perturbing the brain with increasing concentrations of propofol. A recent paper by Lee *et al*. has indeed indirectly suggested that this may be the case.^15^ To test this hypothesis, we reanalyzed EEG data from a previous anesthesia experiment^16^ and compared our results with those from a Kuramoto model. This model consists of partially coupled phase oscillators and has become an archetypal framework to understand brain network synchronization phenomena.^15,17–19^ In order to comprehensively describe the constellation of network-level emergent phenomena seen with propofol, we recorded a continuous high time-resolution evolution of functional connectivity (connectograms) and complexity (complexograms) over all frequencies from 1 to 20 Hz, using four different connectivity metrics spanning time scales from tens of milliseconds to minutes, including phase and power envelope measures, for all topographic scalp regions, and summarized using standard network graph-theoretical indices and a type II complexity measure.

## Materials and Methods

### Data Collection

We reanalyzed a previously collected 31-channel EEG anesthesia dataset recorded from 16 healthy adult volunteers at the University of Oxford in Oxford, United Kingdom (November 11 to 26, 2009). Ethics committee approval and written informed consent had been obtained from all subjects. Volunteers underwent a very slow induction of anesthesia with IV propofol up to an estimated concentration of 4 μg/ml (after 40 min), followed by passive emergence as the propofol concentrations decreased. Time of loss and regain of behavioral responsiveness was assessed by button presses to a cognitive word task every 15 s. Further details of the procedures and changes in EEG slow wave power are described in Mhuircheartaigh *et al.*^16^

### EEG Preprocessing and Analysis

The 31-channel EEG recordings were re-referenced to the average signal and downsampled to 125 Hz. A simple Hjorth-type spatial filter was applied to each electrode’s signal that subtracted the average of the three surrounding electrodes to mitigate the effect of noise, volume conduction, and global common mode signals and enhance the localization of EEG information. The EEG signals were band pass–filtered using third order Butterworth filters with stop-bands 0.75 Hz on either side of every whole number frequency from 1 to 20 Hz. The resulting signals were Hilbert-transformed, and phase and power (in decibels) were extracted for 15-s windows with 5-s overlap.

**Fig. 1.**
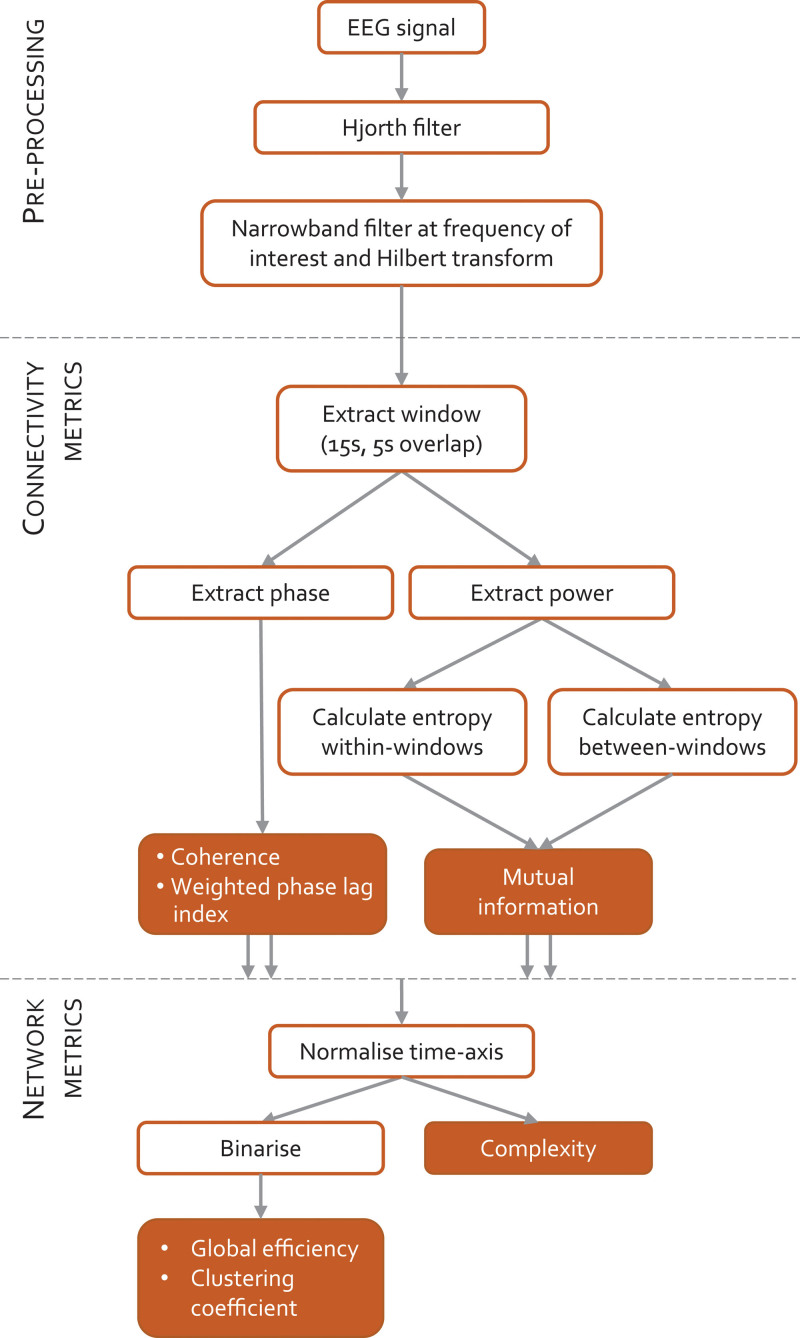
Schematic summary of methodology. Electroencephalogram (EEG) signals from volunteers undergoing a slow induction and emergence from propofol anesthesia are filtered and windowed. Phase and power information are extracted from each 15-s window in order to calculate connectivity with three different metrics. The time axis is then normalized between participants, and the network metrics calculated.

Three types of connectivity metrics were calculated: coherence, weighted phase lag index, and mutual information. Coherence and weighted phase lag indexes are short time scale metrics (less than 5 s) that are sensitive to the phases of individual waves, and mutual information was calculated at two longer time scales that capture the coupling of power envelopes, namely within-window envelope power (medium time scale mutual information) which has a medium time scale (approximately 15 s), and between-window envelope power (long time scale mutual information), which has a long time scale (longer than 60 s). These are EEG metrics chosen to have a similar time scale to those obtained from functional magnetic resonance imaging work. To allow comparison between participants, the time axes of the connectivity time series were normalized to align times of loss and regain of responsiveness. As summary statistics for each functional connectivity metric, we then estimated network global efficiency, clustering coefficient and statistical complexity, focusing on two frequencies of particular interest: 2 Hz (delta range) and 10 Hz (alpha range). A summary of the methods is presented in figure 1.

### Connectivity Metrics

Three functional connectivity metrics were used in this study: coherence, weighted phase lag index, and mutual information. Coherence and weighted phase lag index are phase-based metrics that assess synchrony between EEG channels at specific frequencies. Mutual information is a measure of nonlinear correlation. Connectivity metrics were calculated for 15-s windows with 5-s overlap for each bivariate electrode pair. Since all these metrics are symmetrical, there were 465 unique electrode pairs for our 31-channel EEG dataset.

#### Coherence

Coherence is a measure of how well the instantaneous phases of two signals are phase-locked to each other. If the instantaneous phase of one signal consistently leads or lags that of another signal, the phases are considered locked, and coherence is equal to 1; if the phase lead/lag relationship of two signals is completely independent, coherence will be 0.

#### Weighted Phase Lag Index

The phase lag index measures the extent to which the phase angle differences between two signals are toward the positive or negative sides of the imaginary axis of the complex plane. The “weighted” term refers to how phase differences farther from 0 are weighted more heavily than phase differences close to 0. If all phase angle differences are on the same side of the imaginary axis, the phase lag index will be 1, whereas if the phase lags are evenly spread between the positive and negative sides, the phase lag index will be 0. Volume conduction occurs when two electrodes measure activity from the same source—and therefore will have a phase lag of zero or π—which can make it difficult to interpret the results. To reduce the effects of volume conduction, only nonzero phase lags are considered in the calculation.

#### Mutual Information

Mutual information is a measure of the statistical dependence between signals, sometimes referred to as nonlinear correlation. Signal dependences are assessed by Shannon entropy, which is the amount of information in a variable. Mutual information is then the sum of the entropy in each signal less the joint entropy between the signals. Because critical slowing is the signature of proximity to a phase transition, mutual information was applied to capture network coupling at both medium and long time scales in the EEG power information. *Within-window* mutual information (medium time scale mutual information) used the power changes within each 15-s window, and *between-window* mutual information (long time scale mutual information) used mean power changes over 20 consecutive 15-s windows. The within-window correlation can be thought of as applying the mutual information statistic to the *envelope* of the band pass–filtered EEG signal rather than the signal itself. The between-window correlation quantifies the between-channel mutual information during even longer (205-s) time intervals. Thus, unlike coherence and weighted phase lag index, mutual information is not demonstrating spatial synchrony of individual waves, but instead reflects the processes that drive the slower waxing and waning of EEG power.^20^ It has the disadvantage of a poorer frequency resolution, since envelope fluctuations are mainly generated by underlying broadband processes. The absolute values of mutual information depend on the choice of bin size for the histograms that are used to calculate the various entropies. We took 100 random 2-s samples for each participant’s recording and calculated bin width using the Freedman–Diaconis rule. This has a between-subject normalizing effect.

### Network Metrics

Graph theory is a mathematical framework that describes complex networks as graphs with nodes (also called vertices) and edges (connections between nodes). In brain networks constructed from EEG data, the nodes are the electrodes and the edges are the connectivity values.^21^ Graph theory metrics are most simply applied to binary data (*i.e.*, the presence of a significant connection), which is the approach we take in this paper. We selected the 75th centile of the whole EEG record for each subject as an appropriate threshold for all connectivity metrics. This was chosen as a balance between the extremes of a completely connected network and a completely disconnected network. The results were robust to a range of threshold choices from the 60th to 85th centiles. Two graph theory measures were calculated for each functional connectivity metric: *global efficiency* as a measure of network integration, and *clustering coefficient* as a measure of local efficiency or network segregation. Many other graph theory metrics (such as local efficiency, transitivity, path length, modularity, among others) were found to give similar results to the chosen metrics, and so are not included here. For more information on graph theory metrics, see Tognoli and Kelso.^22^ Note that each metric can be defined for an individual node (electrode, subscript *i*) as well as for the whole network.

#### Global Efficiency

Global efficiency (GE), sometimes called nodal efficiency, is calculated as the mean of the inverse shortest path length from node *i* to all other nodes. A global efficiency of 0 means there are no connections between electrodes; a global efficiency of 1 means every electrode has a direct connection with every other electrode. For a network with N nodes, with d_ij_ as the shortest path length from node *i* to *j*, GE is described by Rubinov and Sporns^23^:


$$GE=\;\frac{1}{n}\underset{i\;\in \;N}{\sum }GE_{i}=\;\frac{1}{n}\underset{i\in N}{\sum }\frac{\sum_{j\in N,\;j\neq i}d_{ij}{}^{-1}}{n-1}$$

#### Clustering Coefficient

The clustering coefficient (CC) of a node measures how many of a node’s connections also have connections with each other. For example, in a social network, if a person’s friends are friends with each other, then they have a high clustering coefficient; if their friends are scattered throughout the world and do not know each other, they have a low clustering coefficient. A high clustering coefficient indicates high *local* efficiency. If *k*_*i*_ is the number of suprathreshold connections for electrode *i*, and *t*_*i*_ is the number of triangles around that electrode, then CC is calculated by


$$CC=\;\frac{1}{n}\underset{i\;\in \;N}{\sum }CC_{i}=\;\frac{1}{n}\underset{i\in N}{\sum }\frac{2t_{i}}{k_{i}(k_{i}-1)}$$

### Complexity

Many differing definitions of complexity have been proposed.^24^ Type II complexities are defined so that the point of maximum complexity lies between pure randomness and rigid order (*i.e.*, complexity is low at both these extremes).^25,26^ A common approach is to estimate the randomness of the system (H) and also the distance (D) of the system from its maximum disorder. This so-called statistical complexity (Cx) is given by multiplying H by D.^27^ We have used normalized Shannon entropy as our measure of randomness (H), and the Jensen–Shannon divergence as disequilibrium (D). Complexity was calculated using each of the four connectivity metrics as inputs.

### Criticality

We used the well-known Kuramoto model of a network of phase-coupled oscillators as a framework to interpret our findings and understand the underlying topology of the brain network dynamics.^28,29^ In particular, it can link changes in connectivity, with complexity and criticality, the three core concepts that underlie our observations. Our model was instantiated in MATLAB (The Mathworks Inc., USA) with code adapted from https://www.math.leidenuniv.nl/scripties/BSC-Zeegers.pdf. It consisted of 500 oscillator nodes, each of whose phase evolves according to its own internal frequency (drawn from a Lorentzian probability density function) and strength of coupling to the other oscillators. The coupling strength was tuned in 50 steps from 0 to 8 to simulate propofol-induced local cortical effects. For each coupling strength, we made 20 runs of 10,000 data points (discarding the first 2,000 points). The output from the model is a time series whose amplitude is dependent on the synchrony of the oscillators—the order parameter. This is analogous to the global efficiency of the EEG network. The sampling rate was arbitrarily set at 100 Hz, which resulted in frequency peaks in the 1 to 40 Hz range, comparable to the EEG. The long time scale mutual information was calculated between windows of 1,000 points lagged by 1,000 time points.

### Statistical Analysis

Unless otherwise stated, data are presented as mean ± SD, and within-subject changes as mean (95% CI) change. All tests were two-tailed with a *P* < 0.05 level of significance. The significance of changes in global efficiency, clustering coefficient, and complexity over time were assessed using repeated measures analysis of variance. Five time segments were chosen that represented each stage of anesthesia: (i) during induction, (ii) just after loss of behavioral responsiveness, (iii) deepest anesthesia, (iv) just before regaining behavioral responsiveness, and (v) during emergence. Each network metric and frequency band was modeled separately. *Post hoc* tests were conducted with Bonferroni correction for multiple comparisons. To establish significance of the loss/regain of behavioral responsiveness peak seen in long time scale mutual information, we applied paired *t* tests between time point (i) and loss of behavioral responsiveness, and between regain of behavioral responsiveness and time point (v). As this was a *post hoc* reanalysis of previously collected data, no formal power analysis was done. However, because this is a study of anesthetic loss of responsiveness (which occurs in every subject), we would expect any putative network indices of loss of responsiveness to show obvious, consistent changes that are present in most of the 16 subjects—and that this number of subjects would enable a reasonable estimation of variance. The EEG data and MATLAB programs are available by contacting the authors.

## Results

### Propofol-induced Changes in Network Coupling

**Fig. 2.**
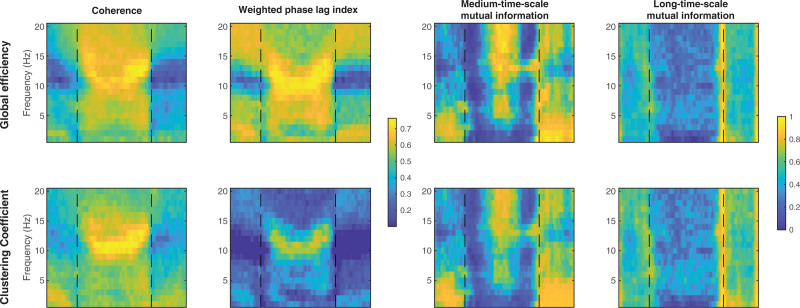
Connectograms of global efficiency (*top row*) and clustering coefficient (*bottom row*) for coherence, weighted phase lag index, medium time scale mutual information, and long time scale mutual information connectivity metrics (*columns*). Connectivity metrics are calculated for each electrode pair to form a network of nodes, and then global efficiency and clustering coefficient summarize an aspect of the network. The mean over all participants (n = 16) is shown here. Loss/recovery of behavioral response are indicated by *vertical dashed lines*. The *x-axis* time scale is standardized such that the loss and recovery of behavioral response for each participant are aligned. The two-dimensional trajectories at 2 and 10 Hz are shown in fig. 3.

**Fig. 3.**
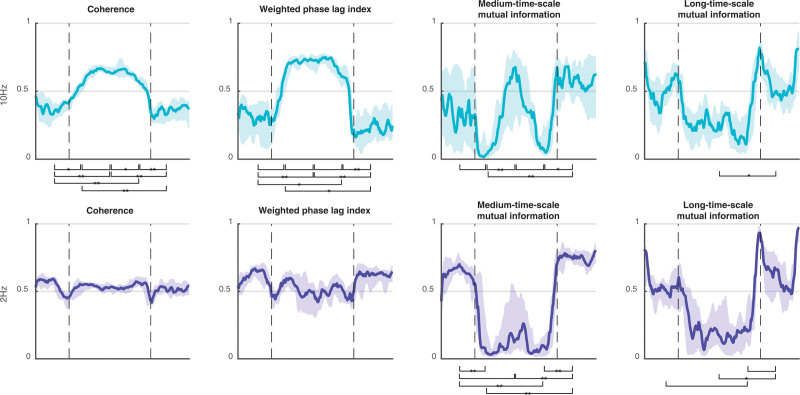
Trajectory of global efficiency (median, 25th to 75th centile) for each connectivity metric at 10 Hz (*top row*) and 2 Hz (*bottom row*). Times of loss and recovery of behavioral response are indicated by *vertical dashed lines*. Significant global efficiency differences between time points i to v (see Materials and Methods) are indicated by a *bracket* (*P* < 0.05), *P < 0.01 and **P < 0.001.

The propofol-induced changes in functional connectivity, as summarized by network metrics and averaged over the 16 subjects (8 female), are presented in table 1, and in figure 2 as “connectograms”—that is, a time against frequency colormap for each different metric, similar to the ubiquitous spectrograms (Supplemental Digital Content 1, http://links.lww.com/ALN/C765, shows the individual connectograms for each subject). The 2-Hz and 10-Hz trajectories from these connectograms, and their significant differences, are shown in figure 3. It is apparent that the pattern of changes in clustering coefficient are like that of global efficiency—with the exception that the clustering coefficient is much lower than global efficiency for the weighted phase lag index connectogram (as might be expected from the aforementioned volume conduction reduction component of its algorithm).

**Table 1.**
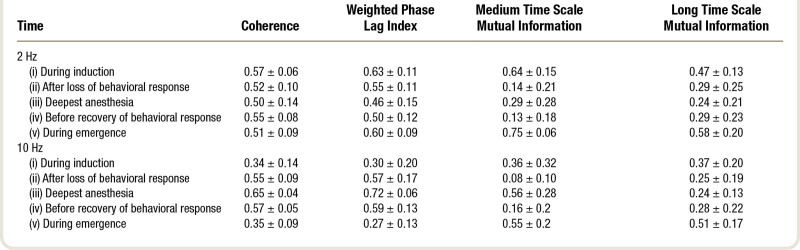
Global Efficiency (Mean ± SD) at Each Time Point for Each Connectivity Metric

The wakeful state was characterized by midrange levels of local and global network efficiency for all functional connectivity. This is consistent with a homogenous and well-integrated network. Around the time of loss of behavioral responsiveness, there were a series of dramatic and complex dynamic changes in connectivity that occurred with relatively small changes in the estimated propofol effect-site concentration—typically over a range of about 0.2 to 0.4 µg/ml. These were mirrored at regain of behavioral responsiveness. However, these changes vary depending on the frequency of interest (Supplemental Digital Content 1, http://links.lww.com/ALN/C765). The statistical significance of changes in global efficiency between time points i to v are indicated visually in figure 3 for 2-Hz and 10-Hz trajectories (*P* values in Supplemental Digital Content 1, http://links.lww.com/ALN/C765).

#### Phase-derived Metrics

**Fig. 4.**
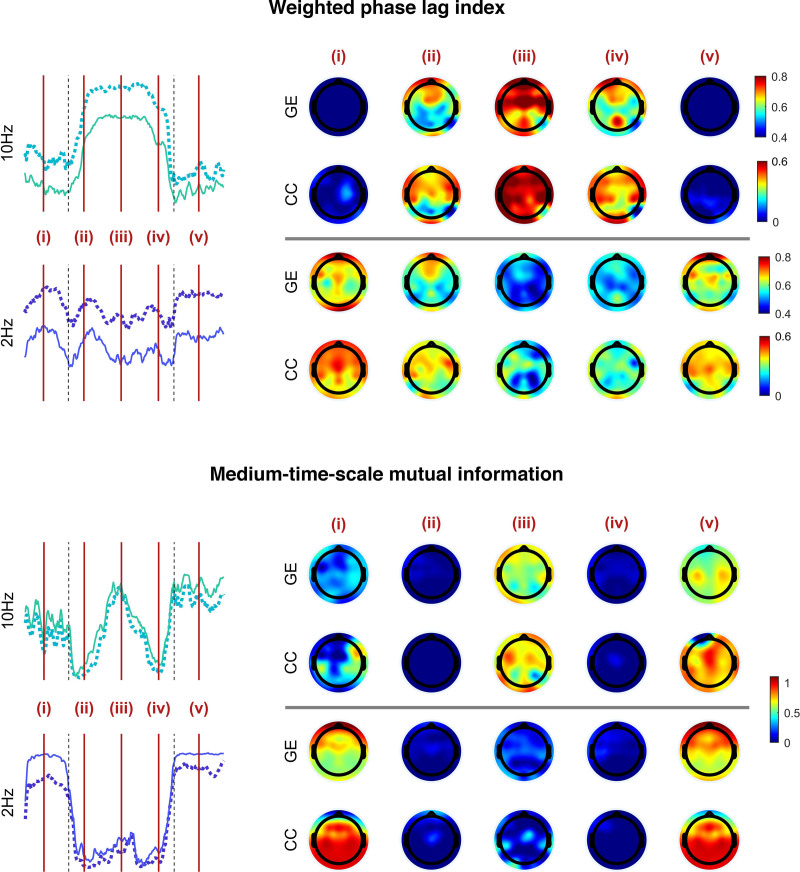
Topographic variation in functional connectivity for weighted phase lag index and medium time scale mutual information for 2 Hz and 10 Hz at time points i through v (see Materials and Methods) Each *headplot* consists of a view from the top of the head, nose upward. The *colors* represent the mean subject global efficiency (GE) and clustering coefficient (CC) calculated for that particular region of the cortex and time point.

Coherence and weighted phase lag index were similar. Low frequencies and very high frequencies (greater than 15 Hz) did not show any significant changes in network efficiency with propofol. Frequencies around 10 Hz (alpha band) showed a significant increase in global efficiency around loss of behavioral responsiveness (mean [95% CI] within-subject increase, 0.27 [0.11 to 0.43], *P* = 0.022 for 10 Hz weighted phase lag index), which further increased (mean [95% CI] additional increase, 0.15 [0.06 to 0.24], *P* < 0.001) to a plateau at peak estimated propofol concentrations (4 µg/ml). During emergence, the pattern reversed, seen as a yellow “smile” in the connectograms (fig. 2, left two columns). As can be seen in figure 4, there were some brain regional differences. The initial increase in 10 Hz weighted phase lag index network efficiency was primarily in the frontal and prefrontal cortices, which then expanded to include the posterior cortices as anesthesia deepened.

#### Power Envelope–based Metrics

The changes in the two mutual information metrics were initially the same for all frequencies. Around loss of behavioral responsiveness, there was a loss of network efficiency in medium time scale mutual information for 10 Hz (mean [95% CI] decrease, −0.28 [−0.45 to −0.11], *P* = 0.032), and a transient peak in long time scale mutual information. However, there was a frequency gradient, where higher frequencies (greater than 10 Hz) transitioned/peaked earlier (*i.e.*, at lower anesthetic concentrations) than the lower frequencies. This is seen as a slight V shape in the induction and emergence transitions in figure 2. During the next few minutes, network efficiency for both medium and long time scale mutual information decreased to almost zero for all frequencies. With further deepening of anesthesia, the network efficiency from medium time scale mutual information then showed a secondary increase for high frequencies (0.48 [0.33 to 0.63], *P* < 0.001, 10 Hz). We speculate that this might be related to the onset of phase-amplitude interfrequency coupling. Network efficiency for all long time scale mutual information frequencies remained low, but with wide interpatient variability. At regain of behavioral responsiveness, long time scale mutual information showed another transient peak for all frequencies, larger than the peak at loss of behavioral responsiveness (*P* < 0.001).

Figure 4 also highlights the fact that propofol reduced the spatial heterogeneity between the local and global efficiencies in the 2-Hz band. In the wakeful state, the global efficiency is maximal in electrodes overlying frontal and prefrontal cortical regions, whereas the clustering coefficient is maximal in posterior regions. Both are lost during unresponsiveness, which is congruent with the previously described observations of loss of long-range frontoposterior communication.^30,31^

In summary, propofol anesthesia was associated with a profound and abrupt *loss* of power-envelope network connectivity for slower frequencies and time scales—but also a coexistent *increase* in alpha frequency network phase coupling. This indicates that the synchrony of the *individual alpha waveforms* in the brain is *increased* by propofol anesthesia, whereas the propofol-induced *decrease* in slow wave synchrony is at the level of the *power envelope* rather than the individual waveforms. The medium-term power envelopes of higher frequencies only start to become synchronous at the highest concentrations of propofol.

### Evidence for Propofol-induced Decrease in Type II Complexity

**Fig. 5.**
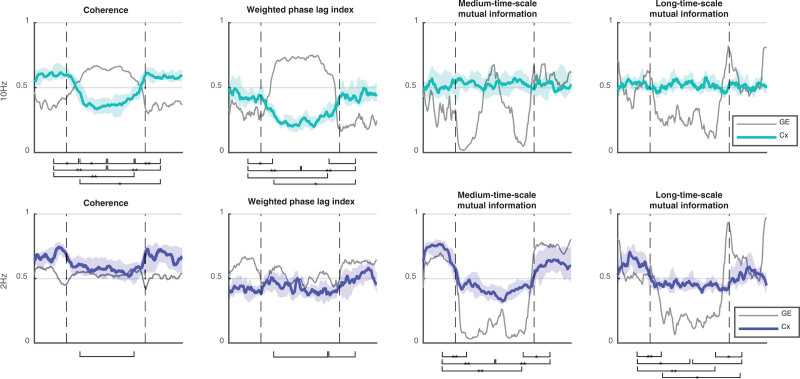
Alpha/delta trajectories of the complexity (*green* and *blue*) with global efficiency in *gray* for comparison. Loss of behavioral responsiveness and regain of behavioral responsiveness are indicated by *vertical dashed lines*. Significant changes in complexity between time points i to v (see Materials and Methods) are indicated with a *bracket* (*P* < 0.05), *P < 0.01 and **P < 0.001.

The changes in the network complexity measure for 2 Hz and 10 Hz are shown in table 2 and figure 5. The previously mentioned *increased* 10 Hz/alpha network phase synchronization after loss of behavioral responsiveness is associated with a concomitant *decrease* in 10 Hz coherence complexity (*P* = 0.005) and weighted phase lag index complexity (*P* = 0.01, fig. 5, row 1, columns 1 and 2). Complexity further decreased at maximum propofol levels (*P* = 0.003). Conversely, the *loss* of power envelope network coupling for 2 Hz after loss of behavioral responsiveness is also seen as a *decrease* in medium time scale mutual information complexity (*P* < 0.001, fig. 5, row 2, column 3). Changes in complexity were minimal for weighted phase lag index at 2 Hz, and for the envelope measures at 10 Hz.

**Table 2.**
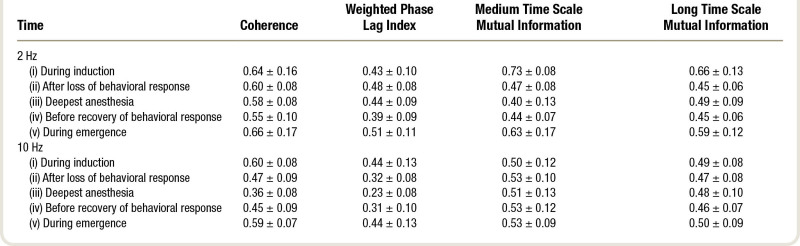
Statistical Complexity (Mean ± SD) at Each Time Point for Each Connectivity Metric

**Fig. 6.**
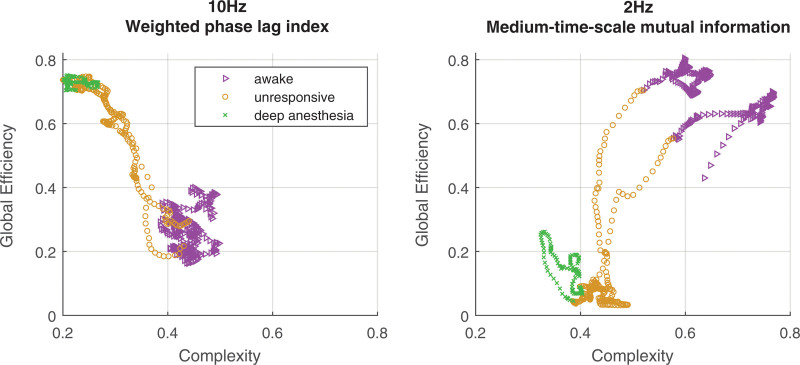
Example of the relationship between complexity and global efficiency. Different *colors* indicate different states of responsiveness.

Figure 6 shows examples of phase plots of complexity against global efficiency. For the 10-Hz frequency, there is a negative correlation between complexity and weighted phase lag index global efficiency, whereas there is a positive correlation with 2-Hz medium time scale mutual information global efficiency.

### Evidence for Propofol-induced Loss of Criticality

Although there are a number of methods that indirectly suggest that the wakeful brain’s network dynamics lie in a region of criticality, proximity to a network phase transition is a pathognomonic diagnostic feature, and our results suggest some sort of network phase transition around loss/regain of behavioral responsiveness. Around loss of behavioral responsiveness, we found a transient increase in long time scale mutual information, followed by abrupt loss of correlation, which we interpret as evidence of the critical slowing phenomenon that is characteristic of proximity to a network phase transition. This loss of correlation for low frequencies and power envelopes coexists with an *increase* in correlation for alpha band wave phase metrics (coherence and weighted phase lag index), indicating a more complex type of phase transition is occurring. This is qualitatively comparable to the archetype of network phase transition phenomena—the Kuramoto model.

**Fig. 7.**
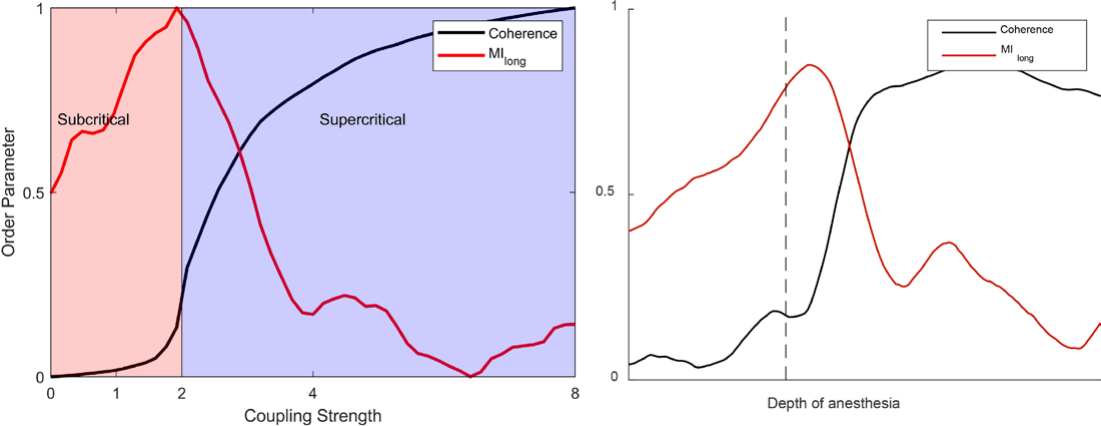
Kuramoto model of network order–disorder phase transition and its subcritical and supercritical phases. The order parameter (coherence) and long time scale mutual information are normalized. The coupling strength is in arbitrary units. The 10-Hz coherence and 2-Hz long time scale mutual information from participant 9 are plotted alongside for comparison.

A typical diagram linking local coupling strength against coherence for the Kuramoto model is shown in figure 7. Of note, a critical point occurs when the local coupling strength has a value of 2. An increase in interoscillator coupling strength beyond this point causes an abrupt increase in the amount of order present in the network—as measured by the increased average coherence of the oscillators (a second-order network phase transition). The global efficiency of the coherence between EEG channels is therefore a plausible, experimentally measurable order parameter (black line). The increase in slow temporal correlation around the critical point is a manifestation of critical slowing in the network, comparable to the between-window mutual information statistics from the experimental data. Even in this simple model, *increased* high frequency oscillator coherence can coexist with *decreased* between-window mutual information (long time scale mutual information, red line)—as observed in our experimental results during the period of propofol-induced unresponsiveness.

## Discussion

The brain is a collaboration of large numbers of neurons and associated cells, so some form of statistical mechanics formulation must apply. In statistical mechanics, the term “phase transition” corresponds to sudden changes of the behavior of systems. In the brain, neuronal-scale effects will drive large-scale network dynamics, but these are not just the summation of the small-scale biologic effects. The observed large-scale changes in brain network connectivity are subject to the conventional equations of dynamics that describe complex systems. In response to increasing concentrations of propofol, we see two clear patterns: the brain appears to simultaneously show alpha wave hypersynchronization and delta power envelope fragmentation, and both the hypersynchrony and fragmentation cause a decrease in network type II complexity. Furthermore, the actual processes of transition to and from unresponsiveness occur abruptly, over only a small change in propofol concentrations, and show patterns that are qualitatively consistent with a network order–disorder phase transition. The biologic significance of this phase transition model lies in the fact that it provides a plausible single unifying explanation for seemingly disparate coupling phenomena. We suggest that this explanation lies at the brain network level. Our results complement previous work (analyzed at the electrode level) suggesting that the transition from consciousness is marked by eigenmodes becoming negative,^14^ reduction in phase lag entropy,^15^ and network hysteresis.^17^ These observations can be understood under the network phase transition framework. In addition, the presence of this nearby phase transition lends support to the idea that the conscious brain functions near, or in, a state of criticality.

The wakeful brain is defined by intermediate and fluctuating levels of regional coupling—indicative that it is lying just subcritical, in a so-called Griffiths phase.^8^ These transient synchronies are manifestations of a metastable system moving between states. A metastable state is characterized by short time-scale phase transitions and lack of a single stable state (*i.e.*, an attractor).^22^ The temporal relationship between behavioral unresponsiveness and the changes in global efficiency is important. Loss of behavioral responsiveness occurred just before, or at the point of, the large-scale network transition. This is consistent with previous functional magnetic resonance work finding that local/fine-scale changes *preceded* global disturbance of network connectivity.^32^ EEG does not have sufficient spatial resolution to confirm this scale sequence of connectivity seen in the functional magnetic resonance imaging, but in general, higher frequencies are associated with smaller spatial scales, and we can clearly see that the transition occurred earlier in the higher frequencies (fig. 2).

### Alpha Wave Band Changes

For frequencies between about 5 and 15 Hz, the most obvious observation is a consistent and progressive increase in global efficiency for the phase-based metrics (coherence and weighted phase lag index) around the time of loss of behavioral responsiveness. In agreement with other work,^33^ the effect started in anterior regions and spread to the rest of the brain with higher propofol concentrations, at which point power-envelope metric synchrony (10 Hz medium time scale mutual information) started to increase too. All nodes became very highly coupled with all other nodes. These observations are not new but have been reported in several previous papers.^6,34^ The mechanisms by which propofol causes these effects are less clear. We would propose that these are the manifestations of generic network-level phenomena that are well established in other fields of network science and percolation theory, and can be described using the conventional existing nomenclature and methods of random network dynamics. Our experimental observations qualitatively follow those seen in the Kuramoto model. Standard network terminology would describe propofol as causing the formation of a single dominant “giant component” in the anterior brain networks.^18^ This has two implications. First, its presence implies the system has crossed a critical threshold and undergone a network phase transition. Second, from the well-known result in the statistical mechanics of complex networks, this is the network analog of a low-temperature Bose–Einstein condensation,^35,36^ where individual nodes lose their independence and function as a single unit—which has obvious implications regarding loss of complexity and information flow. Our findings are in complete agreement with results published by Lee *et al*., who showed a decrease in variation of phase lag (as estimated by its entropy) with propofol-induced unconsciousness (see fig. 3 of their paper).^15^ Additional evidence for the existence of such a network phase transition is the transient increase in long-term variability in global efficiency (time scale of tens to hundreds of seconds), captured by the long time scale mutual information metric. This occurs only around the transition to and from unresponsiveness and is a manifestation of the phenomenon of “critical slowing,” which is pathognomonic of a phase transition.^25^ This is allied with a third indicator, namely hysteresis between losing and regaining consciousness, as has been reported by Huang *et al*. in functional magnetic resonance experiments^37^ and by Warnaby *et al*. in clinical studies.^38^

### Delta Wave Band Changes

Previous work has resulted in conflicting observations as regards the effects of anesthesia on slow wave connectivity. Papers by Bourdillon *et al*.,^39^ and also by Lewis *et al*.,^40^ found fragmentation of slow waves, whereas those by Ma *et al*.^41^ and by Lee *et al*.^42^ found an increase in scalp EEG synchrony. We also did not see much effect from propofol when using the connectivity metrics that quantify the relative phase of individual waves (coherence and weighted phase lag index). However, we did see large decreases during the period of unresponsiveness when using the envelope-based metrics (medium and long time scale mutual information). This agrees with a recently published paper by Duclos *et al*. showing that amplitude-based EEG measures were better able to distinguish levels of anesthesia than phased-based metrics.^43^

### Biologic Explanations and Network Dynamics Explanations

Most previous studies looking at functional connectivity describe the changes in connectivity and what the consequences might be as regards information flux in the brain, but do not address the causes underlying these changes in connectivity. The network dynamics interpretation allows a single unifying explanation for the diverse pattern of connectivity phenomena occurring over a range of different frequencies and time scales. We would propose that the main neuronal effect of propofol is to increase inhibition,^44^ which synchronizes neural assemblies at a small scale (less than 4 cm). This phenomenon has been directly measured *in vivo* and in brain slices^40,45,46^ and is also manifested as the increased scalp EEG alpha and delta wave amplitude seen with propofol. The increased inhibition also causes thalamocortical hyperpolarization *via* a number of cortical and brain stem processes, which results in both thalamic rhythmical burst firing and a thalamocortical feedback loop that predisposes to a 10-Hz resonant frequency.^47,48^ Thus, the increase in local connectivity precipitates a network phase transition that is manifested as the alpha giant component, critical slowing, the delta desynchronization, and the loss of complexity.

### Methodologic Issues

#### Volume Conduction

We reduced volume conduction by using an average reference to minimize common signal effects, and then using a Hjorth derivation to accentuate localization. Although multivariate methods would further reduce volume conduction effects, they do so by completely excluding zero-lag correlations, which would make any giant component invisible. We therefore conducted a sensor-level study and used bivariate analysis methods to quantify within-subject changes.

#### Network Normalization and Surrogate Data

Similarly, it is common practice to compare the experimental data with surrogate randomly phase-shuffled data, to obtain the baseline connectivity distribution that might be observed from an equivalent random network. This is done to determine the statistical threshold for coupling values between particular localized sources. Mutual information from phase-randomized data did not change over the course of the experiment, and thus did not influence our within-subject comparisons.

#### Kuramoto Modeling

This is a simple model that, surprisingly, shows many of the network dynamics that we observed. We employed it because it is a well-accepted way to include dynamics on networks and has a well-understood relationship to mean field cortical modeling. However, future quantitative modeling should involve more complex anisotropic hierarchical network structures that reflect anatomical patterns of structural connectivity, and nonlocal coupling, which can give rise to chimeras—in which the system can transition to situations where synchronous oscillations can co-xist with desynchronized states.^49^ At an individual channel level, our version of the Kuramoto model does not faithfully replicate the details of the EEG spectrograms, and it could be argued that the basic units of the brain are not oscillators. Also, the Kuramoto model does not incorporate any small-scale parameters for neuronal synaptic connectivity or function, so the biologic basis for the “coupling strength” parameter is not explicitly specified.

We conclude that the constellation of alterations in network functional connectivity associated with propofol-induced unresponsiveness are consistent with a complex network order–disorder phase transition of the cortex. Specifically, at concentrations sufficient for unresponsiveness, propofol causes a loss of complexity in cortical network dynamics by inducing a phase transition to a frontocentral 10-Hz hypersynchronous “giant component” and simultaneously causing a profound decrease in the global efficiency of the network in slower time scales. This results in the brain becoming simultaneously too simple and too uncoupled for sufficient information flow required to maintain wakefulness. Measures of brain network dynamics’ proximity to the phase transition could be useful as indicators of imminent transitions in responsiveness.^50^

### Research Support

Dr. Pullon’s salary was funded by a project grant (20/006) from the Australian and New Zealand College of Anaesthetists (West End, Australia), and the James S. McDonnell Foundation (St. Louis, Missouri) grant No. 220023046. This research was funded in part by the Wellcome Trust (grant No. 203139/Z/16/Z) through a contribution to Dr. Warnaby’s salary.

### Competing Interests

Dr. Sleigh serves on the editorial board of Anesthesiology. The other authors declare no competing interests.

## Supplementary Material


