# Supplementary material for: Propofol-induced Unresponsiveness Is Associated with a Brain Network Phase Transition
Source: Anesthesiology. 2022 Feb 8;136(3):420–33. doi: 10.1097/ALN.0000000000004095 (PMC8834166; doi:10.1097/ALN.0000000000004095)

**Supplementary Information**

1. Connectograms for each subject, and each functional connectivity metric. Horizontal axis is normalized time (see text for explanation). The vertical dashed lines are the points of loss and recovery of responsiveness to verbal command. The vertical axes are frequency, and colors depict the strength of the metric at each time point and frequency.

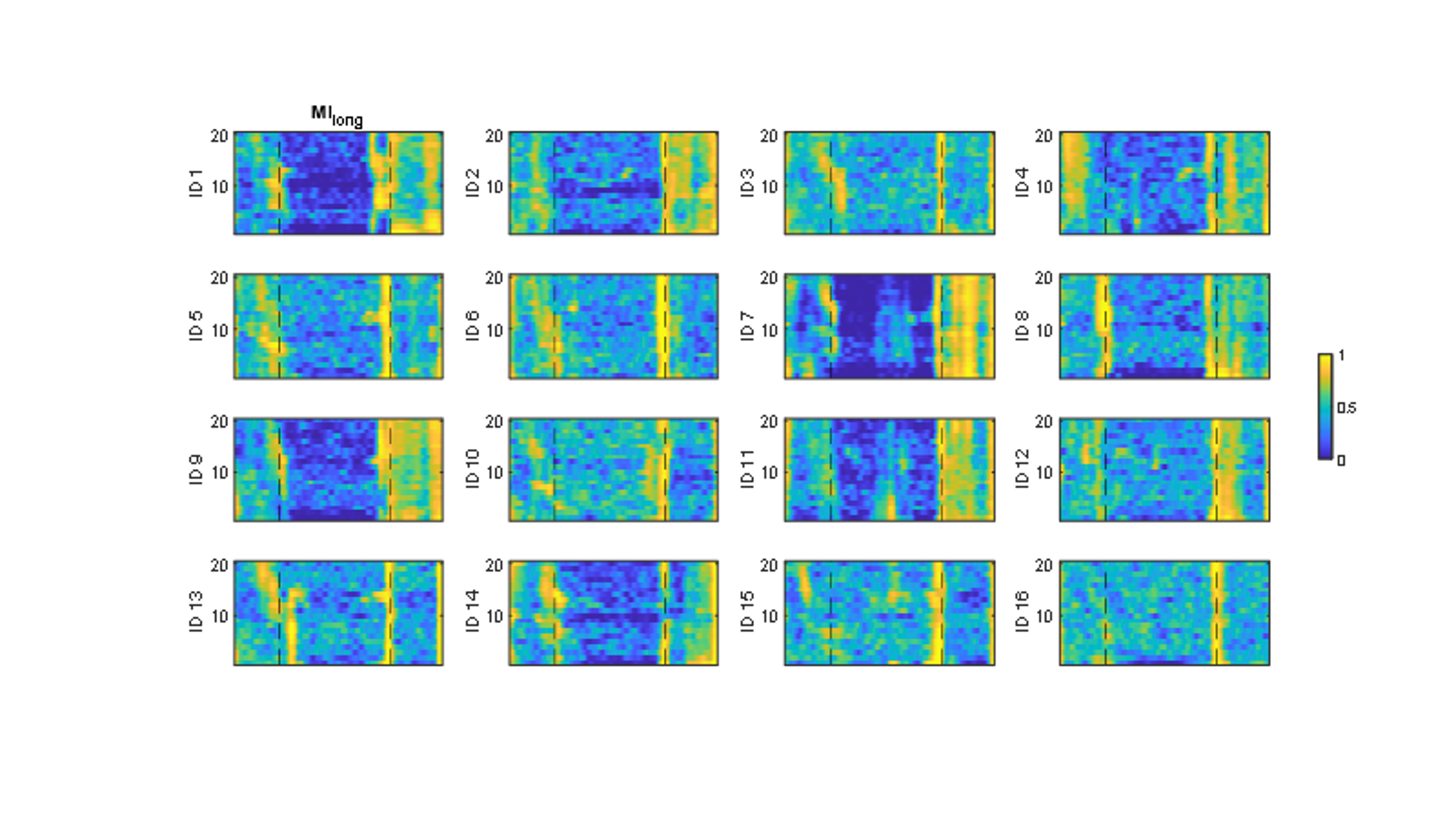


1. Mean [95% confidence intervals] within-subject changes of global efficiency between the different time points, as defined in the Methods section of the paper

**10Hz**

| Time | Coherence | wPLI | MI_medium_ | MI_long_ |
| --- | --- | --- | --- | --- |
| (i) – (ii) | 0.21 [0.12, 0.31] | 0.27 [0.11, 0.43] | -0.28 [-0.45, -0.11] | -0.12 [-0.25, 0.02] |
| (ii) – (iii) | 0.10 [0.05, 0.15] | 0.15 [0.06, 0.24] | 0.48 [0.33, 0.63] | -0.01 [-0.10, 0.07] |
| (iii) – (iv) | -0.08[-0.12, -0.04] | -0.12 [-0.19, -0.05] | -0.40 [-0.62, -0.18] | 0.03 [-0.07, 0.14] |
| (iv) – (v) | -0.22 [-0.27, -0.17] | -0.33 [-0.44, -0.21] | 0.39 [0.20, 0.57] | 0.23 [0.06, 0.41] |

**2Hz**

| Time | Coherence | wPLI | MI_medium_ | MI_long_ |
| --- | --- | --- | --- | --- |
| (i) – (ii) | -0.05 [-0.11, 0.02] | -0.08 [-0.18, 0.02] | -0.50 [-0.63, -0.38] | -0.19 [-0.32, 0.06] |
| (ii) – (iii) | -0.02 [-0.08, 0.03] | -0.09 [-0.18, 0.00] | 0.15 [-0.06, 0.36] | -0.05 [-0.19, 0.10] |
| (iii) – (iv) | 0.05 [-0.01, 0.10] | 0.04 [-0.04, 0.11] | -0.15 [-0.36, 0.06] | 0.05 [-0.11, 0.20] |
| (iv) – (v) | -0.04 [-0.13, 0.05] | 0.11 [0.00, 0.22] | 0.62 [-0.51, 0.73] | 0.29 [0.10, 0.48] |


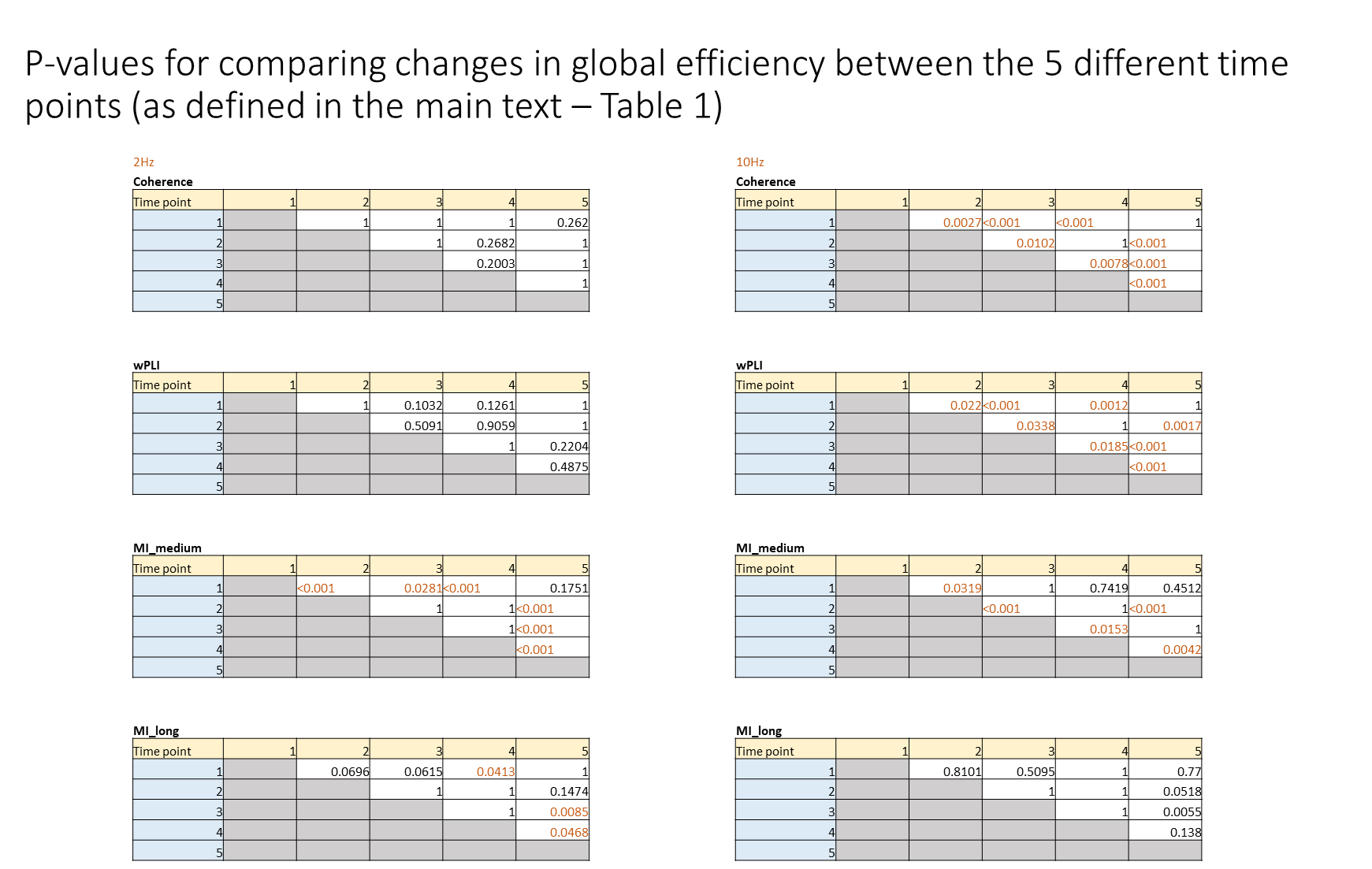


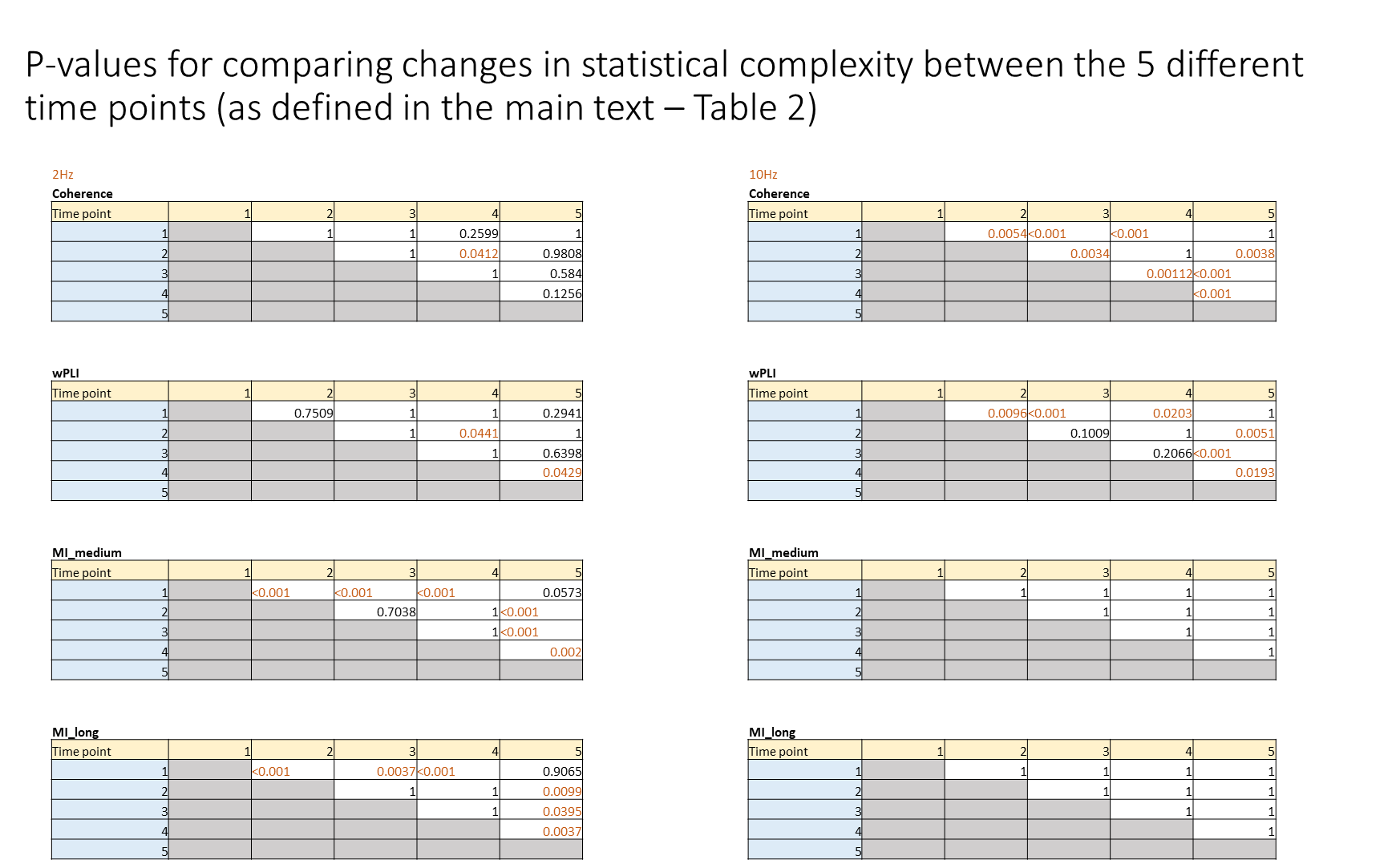

Supplement: Supplementary file 1 [file aln-136-420-s001.docx]
